# Automated Deep Learning-Based Demyelination Load Segmentation in Metachromatic Leukodystrophy

**DOI:** 10.1007/s00062-026-01652-6

**Published:** 2026-04-21

**Authors:** Pascal Martin, Joël Schaerer, Thomas Cajgfinger, Alessandro Delmonte, Benjamin Bender, David Whiteman, C.J. Malanga, Alexandre Fusellier, David Scott, Joyce Suhy, Hendrik Rosewich, Samuel Groeschel, Luc Bracoud

**Affiliations:** 1 Department of Neurology and Epileptology, Hertie Institute for Clinical Brain Research, University of Tübingen, Tübingen, Germany; 2 Clario, Inc. (formerly Bioclinica, Inc.), Lyon, France; 3 Department of Diagnostic and Interventional Neuroradiology, University of Tübingen, Tübingen, Germany; 4 Takeda Pharmaceutical Company Ltd, Cambridge, MA, United States; 5 Clario, Inc. (formerly Bioclinica, Inc.), San Mateo, CA, United States; 6 Department of Pediatric Neurology & Developmental Medicine, University Children’s Hospital Tübingen, Tübingen, Germany; 7 Department of Pediatric Neurology, University of Göttingen, Göttingen, Germany; 8 Experimental Pediatric Neuroimaging, University Children’s Hospital Tübingen, Tübingen, Germany

**Keywords:** Metachromatic leukodystrophy, Convolutional neural network, Demyelination load, Quantitative MRI, Automated lesion segmentation

## Abstract

**Purpose:**

Metachromatic leukodystrophy (MLD) is a rare lysosomal storage disorder characterized by progressive white matter demyelination. Quantification of demyelinated white matter on MRI—typically expressed as the *demyelination load*—serves as a key imaging biomarker of disease burden, enabling objective monitoring beyond visual rating scales. However, current semi-automated pipelines are limited by manual interaction, pediatric brain variability, and differences in MRI acquisition. This study aimed to develop and validate a self-configuring convolutional neural network (CNN) for automated segmentation of demyelinated white matter in MLD and to compare its performance with a conventional semi-automated method across heterogeneous MRI datasets.

**Methods:**

An nnU-Net was trained on 189 3D T1- and axial T2-weighted scans from 35 MLD patients using visually controlled conventional masks as ground truth. Independent testing was performed on 130 scans (73 high-resolution 3D, 57 lower-resolution 2D T1-weighted) from 49 patients. Performance was assessed by Dice coefficient, Bland-Altman bias, correlation with Gross Motor Function Classification (GMFC-MLD), MLD MRI severity score, longitudinal consistency, and qualitative review of outliers.

**Results:**

CNN-based segmentation showed strong spatial agreement with the reference method, with a median Dice coefficient of 0.82 for 3D T1-weighted scans and 0.75 for 2D scans. Volumetric bias was minimal on Bland-Altman analysis. CNN-derived demyelination load correlated significantly with motor impairment (r_S_ = 0.38 for 3D and r = 0.56 for 2D; both *p* < 0.001) and showed a stronger association with the MLD MRI severity score than conventional segmentation (3D: r_S_ = 0.48 vs. 0.28; 2D: r_S_ = 0.83 vs. 0.29). Correlations with clinical status were slightly lower (CNN: r_S_ = 0.38, *p* < 0.001; conventional: (r_S_ = 0.26, *p* < 0.025)) Longitudinal analyses demonstrated stable, monotonic changes over time, and qualitative review revealed fewer boundary misclassifications.

**Conclusion:**

The nnU-Net enables fast, reproducible, and clinically meaningful segmentation of demyelinated white matter in MLD. It generalizes across MRI protocols, correlates with motor function, and offers a scalable tool for standardized biomarker extraction in clinical trials and other leukodystrophies.

## Introduction

Metachromatic leukodystrophy (MLD) is a rare autosomal-recessive lysosomal storage disorder caused by mutations in the *ARSA* gene, leading to deficient arylsulfatase A activity and resulting in sulfatide accumulation. This pathological cascade culminates in progressive demyelination of central and peripheral nervous systems, clinically presenting with motor and cognitive decline [1–3].

MRI plays a pivotal role in diagnosing and monitoring MLD. T2-weighted hyperintensities, often exhibiting a tigroid pattern, are the hallmark imaging feature [2, 4]. To quantify disease burden, several imaging-based approaches have been developed. The most straightforward method is the MLD MRI severity score [5], a visual rating scale assessing the extent of white matter lesions and global brain atrophy across predefined regions. While widely used in clinical routine, this approach remains subject to inter-rater variability and lacks more detailed quantification of T2-hyperintensities. To reduce subjectivity and enable objective, quantitative assessment, the “demyelination load” was introduced [6]. This method semi-automatically quantifies the proportion of T2-hyperintense white matter relative to total brain volume, allowing reproducible estimation of disease burden validated in landmark studies [2, 7–9] and has become a reference standard in MLD imaging.

The demyelination load method relies on tissue segmentation using standard MRI volumetry pipelines such as SPM12 and CAT12 to generate tissue probability maps. These maps are then used to define white matter regions, within which abnormal T2 signal intensity is segmented and quantified. While robust, the method carries limitations: segmentation accuracy may be reduced in a disease affected pediatric population due to anatomical variability and atrophy, brain maturation effects, scanner-dependent image quality. Furthermore, conventional segmentation algorithms typically rely on adult-derived tissue probability maps, which may inadequately represent age-specific anatomical features; this discrepancy, compounded by disease-related structural alterations, further undermines tissue classification accuracy [10, 11]. Thus, while the technique is semi-automated and reproducible, it remains constrained by methodological assumptions.

To overcome these limitations and improve efficiency, machine learning approaches—particularly deep learning—have emerged as powerful tools for white matter lesion segmentation. Previous work has demonstrated their utility in adult populations with multiple sclerosis or cerebral small vessel disease, achieving high accuracy and reliability [12–14]. However, applications in pediatric leukodystrophies, and in particular in MLD, are missing.

In this study, we trained a convolutional neural network (CNN) on a curated dataset of MLD patients for whom demyelination load was previously calculated using high-resolution 3D T1-weighted images and axial T2-weighted sequences. The ground truth segmentations were derived using the established semi-automated pipeline with strict visual quality control. We then tested the model on an independent dataset including both high-resolution 3D and 2D T1-weighted images, each paired with T2 images, to assess generalizability. Evaluation metrics included Dice coefficient (DC) and Bland-Altman analysis, alongside correlation with clinical measure of motor symptoms and longitudinal evaluation. Our goal was to assess whether this CNN-based method can serve as a robust alternative to the conventional pipeline, facilitating more efficient and standardized lesion quantification in MLD.

## Materials and Methods

### Study Design and Cohorts

This study followed a two-phase design involving a training and a test cohort in MLD patients. MLD diagnosis was confirmed based on deficient arylsulfatase A (ARSA) enzyme activity, elevated urinary sulfatides, clinical presentation, and (where available) pathogenic mutations in the *ARSA* gene. Ethical approval was obtained from the Ethics Committee of the University of Tübingen (reference 401/2005), and written informed consent was provided by patients or legal guardians.

### Imaging Data and Preprocessing

All MRI scans included both T1-weighted and T2-weighted sequences. Data were acquired across multiple clinical sites using scanners from different manufacturers (GE, Philips, and Siemens) and at field strengths of 1.5 T and 3 T, reflecting real-world heterogeneity in acquisition protocols. This diversity was considered advantageous for developing a robust and generalizable segmentation approach.

Visual quality control (QC) was applied to all images. T1- and T2-weighted scans were manually inspected for motion artifacts and insufficient brain coverage. Scans with substantial technical limitations were excluded.

### Conventional Demyelination Load Segmentation

The reference standard for both CNN training and evaluation was derived using a semi-automated lesion segmentation method validated in previous studies [6, 7]. The pipeline was applied to both training and test data as follows:

- T2-weighted images were rigidly co-registered to corresponding T1-weighted images.
- Tissue segmentation was performed using the CAT12 toolbox within SPM12, generating grey matter, white matter, and CSF probability maps based on adult tissue priors.
- White matter abnormalities were identified by modeling the T2 intensity distribution within the white matter mask using Gaussian mixture modeling.
- Lesion voxels were defined by thresholding at the intersection of Gaussian components and refined using a Markov Random Field (MRF) algorithm to reduce noise.
- Voxels in clusters < 200 were excluded to minimize false positives.

All resulting segmentations were visually inspected. For the training dataset, conventional segmentations were visually reviewed and manually corrected where minor changes were necessary to obtain high-quality reference masks suitable as ground truth for CNN training. Scans with grossly incorrect or anatomically implausible masks were excluded from CNN training.

### CNN-Based Segmentation

**Fig. 1:**
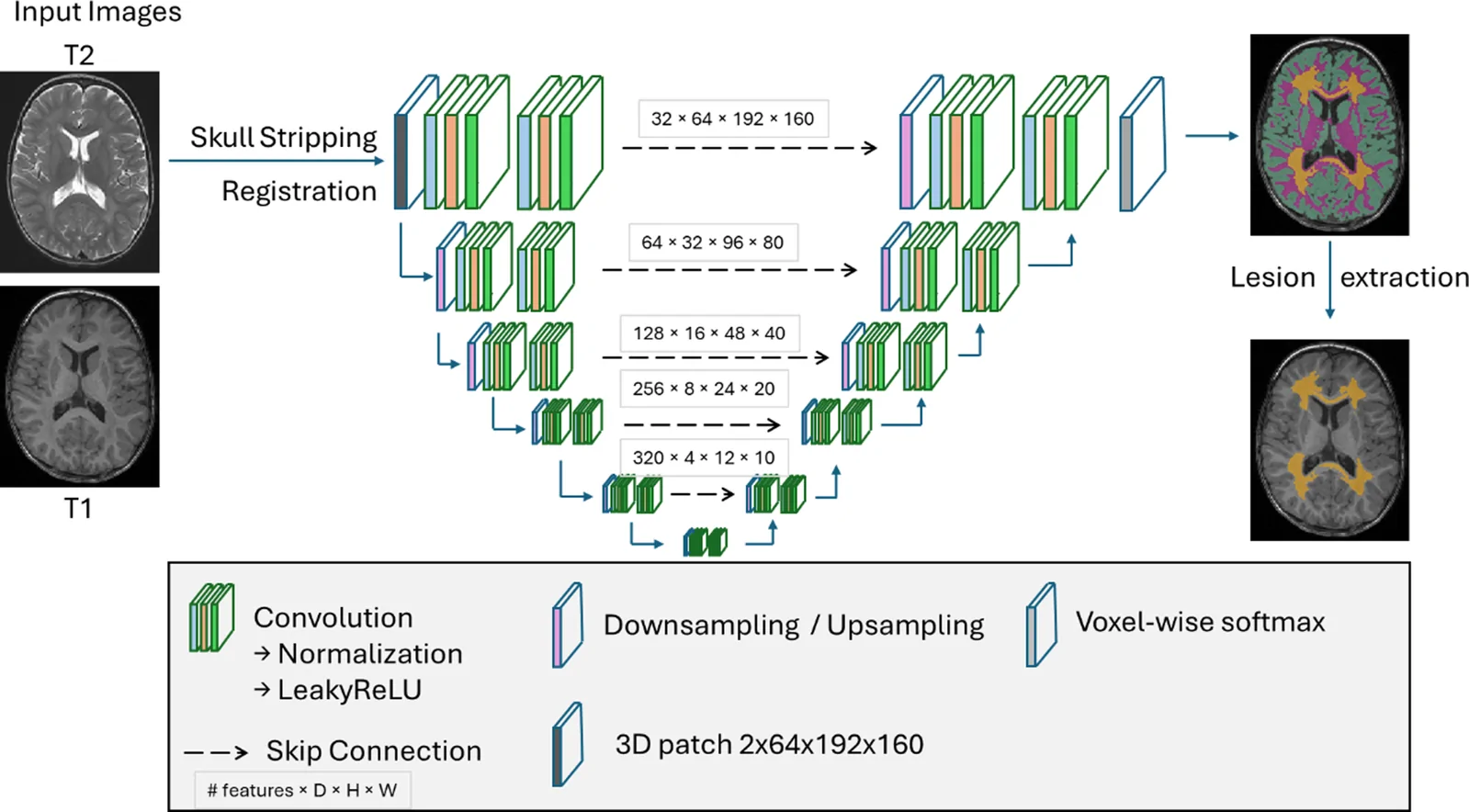
Architecture of the implemented nnU-Net model for MLD lesion segmentation*. *(Schematic overview of the nnU-Net pipeline used for automated demyelination load segmentation. The proposed model is based on a 3D U‑Net architecture processing input patches of size 64 × 192 × 160 voxels. Co-registered and skull-stripped images are concatenated and used as input to the first layer of the model (dark gray). The 6‑level encoder-decoder module with skip connections (dotted arrows) enforces multi-resolution image feature learning and it was trained using a weighted Dice and cross-entropy loss function. Each level implements a series of convolutions with a 3 × 3 × 3 kernel, normalization and LeakyReLU activations. The final softmax layer (light gray) assign voxel-wise probabilities to each of the three output classes, including MLD lesions, healthy white matter and gray matter. Joint learning of lesion and healthy brain tissue proved beneficial in model convergence. The final MLD lesion mask is computed and binarized from post-processed softmax probabilities.)

A 3D U‑Net model based on the nnUnet framework [12] was trained to jointly segment MLD white matter lesions, gray matter, healthy white matter and cerebrospinal fluid (CSF). The architecture follows an encoder-decoder architecture with six layers combining a series of 3D convolutions, instance normalization and LeakyReLU activation layers (see Fig. 1). Co-registered T1 and T2-weighted images were used as input to the model after resampling 0.5 × 0.5 × 1.2 mm and random 3D patch cropping with size 64 × 192 × 160 vox.

The model was trained using 5‑fold cross-validation, implementing extensive data augmentation to address the limited dataset size. Random data augmentation included flipping, rotation, scaling and intensity-based transformations. All folds were trained on a NVIDIA A10G GPU using the Adam optimizer with a batch size of 2.

. Predictions from the five cross-validated models were ensembled, averaged and applied to the independent test set (Group 2), which included both high-resolution 3D T1 and lower resolution 2D T1 images. The ability of the CNN to generalize across scan types was a critical component of the evaluation.

Following each segmentation process, the output maps generated by both the CNN and the conventional method were reviewed visually to ensure technical validity. Specifically, the alignment of T1 and T2 images, the plausibility of grey and white matter classification, and the anatomical consistency of lesion masks were assessed. Outputs with clear technical errors in the conventional segmentation approach (e.g., misregistration or incomplete segmentation) were excluded from subsequent analyses. Importantly, this procedure did not involve excluding cases based on prediction quality, but solely to remove technically invalid results that would preclude meaningful evaluation and did not include manual correction or refinement.

### Patient Cohort

This study included two independent datasets: a training dataset and a test dataset. To avoid overlap of patients between the training and test groups, MRI scans were recruited from separate projects. Specifically, the training cohort consisted of data from a previously published study [15], whereas the test dataset was collected independently at the Department of Diagnostic and Interventional Neuroradiology, University Hospital Tübingen. Due to this approach, although similar, exact matching for age or gender between the two datasets could not be ensured.

#### Training Dataset

Initially, 221 MRI scans from 44 individual patients (20 female; median age 4.3 years, range 1.7–10.9 years) were included. Following rigorous visual quality control (QC), 189 MRI scans from 32 patients remained (14 female; median age 4.2 years, range 1.7–10.9 years; mean number of scans per patient: 4). For model training, a particularly stringent QC threshold was applied to ensure optimal input quality and minimize noise in the ground truth segmentations. Both T1- and T2-weighted images were required to be free of relevant motion artifacts, as high-quality, co-registered input from both modalities was essential for subsequent automated processing. In some cases or in settings where sedation was minimized for clinical reasons, residual motion led to consistently reduced image quality. Consequently, all scans from certain subjects had to be excluded when neither scan met the predefined quality standards. These exclusions reflect the practical challenges of pediatric MRI acquisition rather than site-specific shortcomings and ensured that only technically reliable data contributed to model training.

#### Test Dataset

The test dataset initially consisted of 196 MRI scans from 49 individual patients (23 female; median age 13.0 years, range 1.4–33.4 years). Following visual quality control, 130 MRI scans from 42 individual patients remained (26 female; median age 12.4 years, range 1.5–33.7 years; mean number of scans per patient: 4).

An overview is presented in Table 1.

**Table 1:** Overview of the patient cohorts included in the training and test datasets before and after visual quality control (QC).

Dataset	Initial scans (patients)	After QC scans (patients)	Female	Median age (range)	Mean scans per patient
Training dataset	221 (44)	189 (32)	14	4.2 (1.7–10.9)	4
Test dataset	196 (49)	130 (42)	26	12.4 (1.5–33.7)	3

The test dataset was further subdivided into two subgroups based on imaging protocols:

- 73 scans from 31 individual patients (14 female, median age 13.5 years, age range 1.5–33.4 years) with high-resolution 3D T1-weighted gradient-echo (GRE)sequences (median resolution 1 × 0.97 × 1 mm^3^) and matching T2 axial images (median resolution 0.5 × 0.5 × 3.3 mm^3^)—hereafter referred to as 3D T1-based group. These 3D gradient-echo acquisitions represent the high-resolution standard typically used in research and advanced clinical protocols and provide a consistent imaging basis for quantitative analysis.
- 57 scans from 24 individual patients (12 female, median age 9.7 years, age range 2.0–23.1 years) with lower-resolution 2D T1-weighted turbo spin echo (TSE) sequences (median resolution 0.66 × 0.74 × 5 mm^3^) and T2-weighted axial images (median resolution 0.5 × 0.5 × 5.3 mm^3^)—hereafter referred to as 2D T1-based group. The inclusion of T1-weighted TSE sequences, as opposed to the 3D gradient-echo acquisitions used in the other subgroup, reflects real-world variability in clinical MRI protocols and allows assessment of model generalizability across distinct acquisition settings.

Importantly, the CNN was trained only on 3D MPRAGE data but tested on both 3D and 2D T1-weighted inputs to assess generalizability.

### Evaluation Strategy

To comprehensively assess CNN performance, we applied both quantitative and qualitative metrics. The conventional method was applied to the test cohort using the same pipeline described above. Comparative analyses were performed on the CNN and conventional segmentations:

1. Spatial Overlap: Dice coefficient (DC) was calculated to measure spatial agreement between CNN and conventional outputs.
2. Volume Agreement: Bland-Altman analysis was used to assess volumetric differences between methods.
3. Outlier Analysis: Dice and Bland-Altman outliers were reviewed visually. These cases were evaluated across scanners, age groups, and anatomical variance (e.g., unmyelinated regions or enlarged ventricles) to determine which segmentation method better handled these conditions.
4. Clinical Correlation: Lesion volumes were correlated with Gross Motor Function Classification for MLD (GMFC-MLD) using Spearman’s rank correlation.
5. Longitudinal Stability: For patients with multiple scans, lesion volume change between consecutive timepoints was measured to evaluate consistency and plausibility.

For the calculation of the *demyelination load* (white matter lesion volume divided by total brain volume), the total brain volume was defined as the sum of gray matter and total white matter (normal-appearing white matter and white matter lesion volumes). Importantly, total brain volume was derived separately within each segmentation pipeline—using SPM-based tissue maps for the conventional approach and CNN-derived tissue classes for the deep learning approach—to ensure internal consistency of both methods.

This multi-pronged strategy allowed evaluation not only of segmentation accuracy but also of clinical relevance and robustness to structural variability, especially in a pediatric rare disease context.

## Results

### Segmentation Performance

**Fig. 2:**
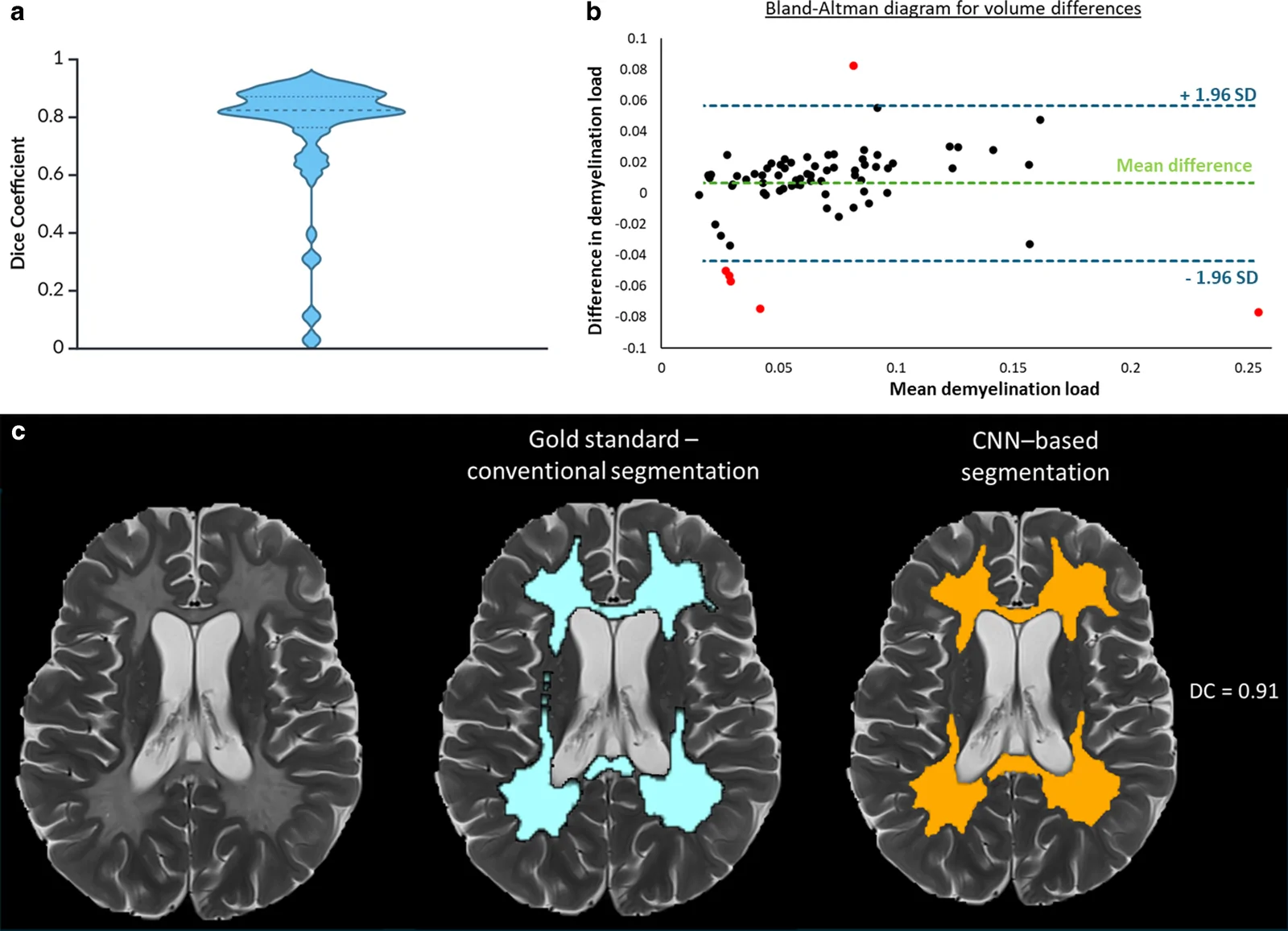
Performance of the CNN-based lesion segmentation. (**a**) Distribution of Dice coefficients across all 3D T1-aquised test cases (*n* = 73) for the automated CNN-based segmentation of demyelinating lesions. The width of each violin corresponds to the frequency of Dice scores; dashed lines indicate the median (0.82) and interquartile range,. (**b**) Bland-Altman diagram depicting agreement between CNN-based and conventional segmentation methods for estimating demyelination load. Each point represents the difference in demyelination load estimates plotted against their mean. For 3D scans, 6 out of 73 cases (8.2%) fell outside the 95% limits of agreement, with the CNN-based model yielding slightly higher demyelination load (median difference: +0.0115). No systematic bias between the two methods was observed. Outliers predominantly occurred in scans with low lesion burden. (**c**) Representative segmentation example with a very good dice coefficient of 0.91 (left—Original T2-weighted image; middle—conventional segmentation of demyelination load projected onto the T2 image (turquoise); right—CNN-based segmentation of demyelination load (orange))

The CNN-based model demonstrated good agreement with the conventional demyelination load segmentation method. The median Dice Coefficient (DC) across all 73 3D T1-based scans DC reached 0.82 (SD = 0.21). As shown in Fig. 2, the violin plot of Dice coefficients is tightly concentrated in the 0.8–0.9 range, reflecting consistently high overlap between CNN and conventional segmentations for the vast majority of cases. A small number of points extend well below this band, however, representing rare outliers (Dice scores down toward 0.2–0.4), which were evaluated visually in the outlier analysis.

Bland-Altman analysis demonstrated low bias between the two segmentation methods. For 3D T1-images, 6 out of 73 cases (8.2%) fell outside the limits of agreement with CNN-based model estimating a slightly higher demyelination load than the conventional segmentation method (median +0.0115).

### Correlation with Clinical Parameters, MLD MRI Severity Score and Longitudinal Consistency

**Fig. 3:**
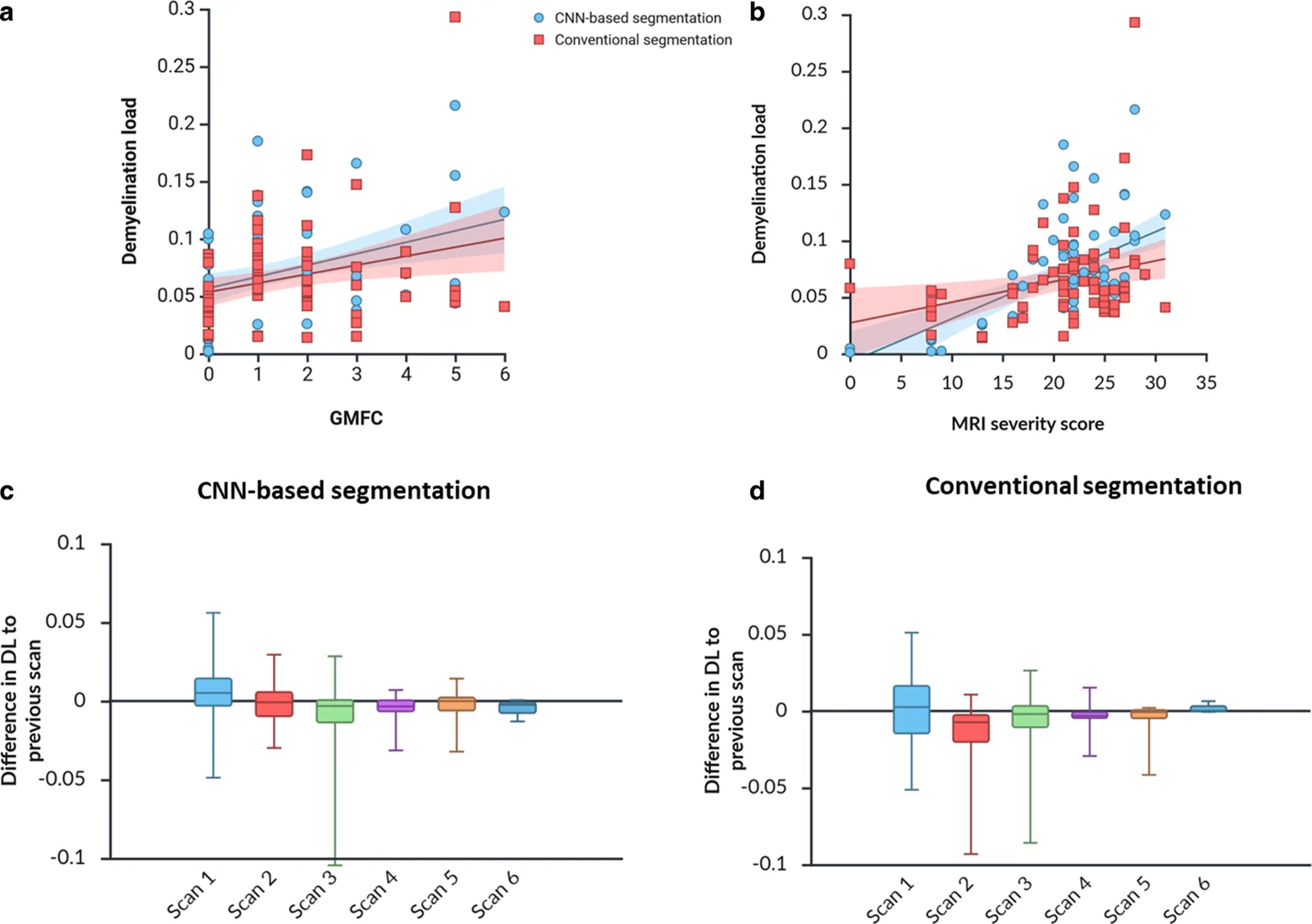
Correlation of demyelination load with clinical and radiological severity and longitudinal consistency of demyelination load changes across consecutive scans. (**a** Scatter plots illustrating the relationship between clinical motor impairment (GMFC score at the time of MRI) and demyelination load derived from CNN-based (blue circles) and conventional (red squares) segmentation methods. Shaded areas indicate the respective 95% confidence intervals. Demyelination load correlated significantly with GMFC score for both methods, with a stronger association for CNN-based segmentation (Spearman’s r = 0.38, *p* < 0.001) than for conventional segmentation (r = 0.26, *p* = 0.025),** b** Scatter plots showing the relationship between MLD MRI severity score and demyelination load for the 3D T1-weighted cohort. Demyelination load derived from the CNN showed a stronger correlation with MLD MRI severity score (Spearman’s r = 0.48, *p* < 0.001) compared with the conventional approach (r = 0.28, *p* = 0.017),** c–d** Box-and-whisker plots illustrating longitudinal changes in demyelination load between consecutive MRI time points across patients with serial imaging. Each data point represents the difference between two consecutive scans of an individual patient (later minus earlier time point). Predominantly negative values reflect the expected increase in demyelination load over time. Panel C shows CNN-based results, Panel D shows conventional segmentation. Both methods demonstrated stable and clinically plausible longitudinal behavior, with only few outliers, supporting their suitability for longitudinal monitoring.)

Demyelination load derived from the CNN model correlated significantly with clinical severity (see Fig. 3). GMFC scores at the time of MRI demonstrated a significant positive association with lesion load as identified by the CNN-based model (r_S_ = 0.38, *p* < 0.001), suggesting that higher motor impairment corresponded to a greater extent of demyelination. The conventional segmentation yielded a statistically significant correlation as well, which was somewhat weaker than that of the CNN-based segmentation (r_S_ = 0.26, *p* < 0.025); however, the difference did not reach statistical significance (Williams’ test: t = 1.81, *p* = 0.075). For comparison, the semiquantitative MLD MRI severity score showed a slightly stronger correlation with GMFC (r_S_ = 0.42, *p* < 0.001).

In patients with multiple longitudinal datasets, the median change in demyelination load between consecutive time points showed a slight progression consistent with the expected disease course. This trend was observed in both the CNN-based and the conventional segmentation approaches. Apart from a few outliers, no systematic implausibilities were detected in either method.

### Outlier Analysis

**Fig. 4:**
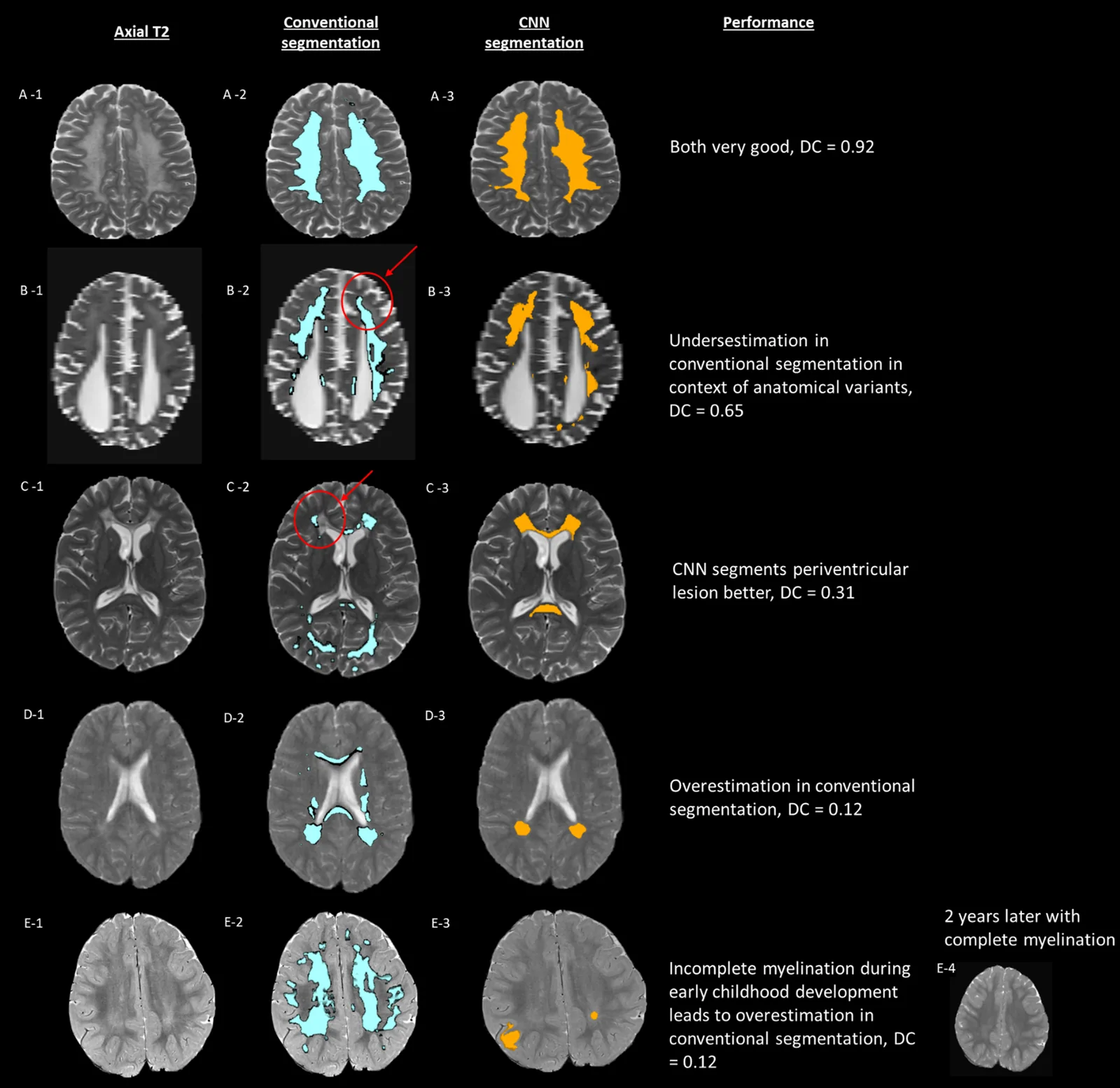
Representative cases with low and high Dice coefficients illustrating differences between CNN-based and conventional segmentation Examples **a**–**e** illustrate cases with high (**a**) and low (**b**–**e**) Dice coefficients between conventional and CNN-based segmentation of T2-hyperintense white matter (demyelination load). Each case is presented in three columns: left—axial T2-weighted image, middle—conventional segmentation of demyelination load projected onto the T2 image (turquoise)—right—CNN-based segmentation overlaid projected onto the T2 image (orange). (**a**) Depicts a case with the highest Dice coefficient (0.92), showing close visual correspondence between CNN-based and conventional segmentation, with no evident discrepancies. (**b**) A case with ventricle deformation and relatively low-resolution T2 imaging, where the extent of frontal white matter abnormalities is underestimated in the conventional segmentation. (**c**) Demonstrates undersegmentation by the conventional method near the frontal horn of the lateral ventricle, where adjacent hyperintensities are incompletely captured. (**d**) Shows spurious lesion detection in the conventional segmentation, especially in periventricular regions and the corpus callosum, which are not supported by visual T2 hyperintensities. (**e**) The case with the lowest Dice coefficient, obtained in a patient aged 1.4 years. Conventional segmentation overestimates lesion volume, likely due to misclassification of incompletely myelinated regions as lesions. Panel E‑4 (same patient at age 3.4 years) shows nearly complete myelination with only subtle parietal T2-hyperintensities, likely reflecting early pathology. On the other hand, the CNN-based segmentation reveals a misclassification of gray matter as white matter lesion

**Fig. 5:**
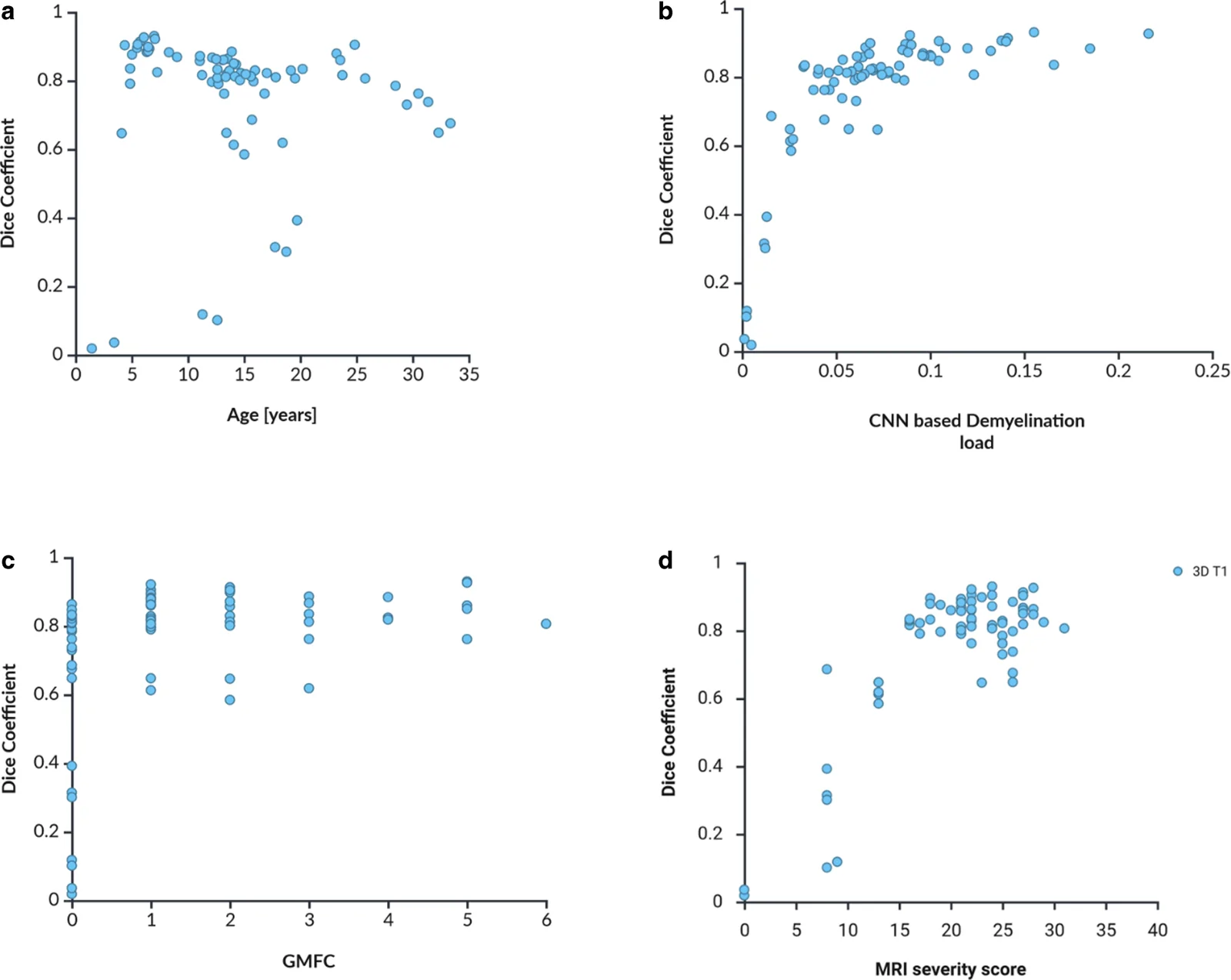
Dice coefficient between CNN-based and conventional segmentations across clinical and radiological parameters Comparison of segmentation overlap (Dice coefficient) between the CNN-based model and conventional SPM-based segmentation for T2-hyperintense white matter (demyelination load) across different clinical and radiological parameters. Each subplot shows the Dice coefficient as a function of one of the following variables: (**a**) patient age at scan, (**b**) CNN-based demyelination load, (**c**) GMFC score at scan time, and (**d**) MLD MRI severity score. Higher Dice values indicate better agreement. Lower segmentation concordance was observed in younger patients and in those with lower lesion burden, suggesting greater variability or structural immaturity in early disease stages

Qualitative review of individual cases with low Dice scores or outliers in the Bland-Altman diagram revealed that discrepancies clustered in few individuals where misclassification arose from partial volume effects especially near the ventricles/CSF, overinterpretation of incomplete myelination in young patients or altered anatomy with expanded ventricles or advanced atrophy. In all cases, the CNN approach better matched the visual extent of demyelination, particularly in scans with lower contrast. In cases of extensive white matter alterations, both methods were able to accurately capture the extent of the changes, demonstrating a high DC and strong visual concordance (see Fig. 4). Low values of each demyelination load, MLD MRI severity score, age and GMFC were associated with lower agreement (see Fig. 5).

## Comparison Across Modalities

**Fig. 6:**
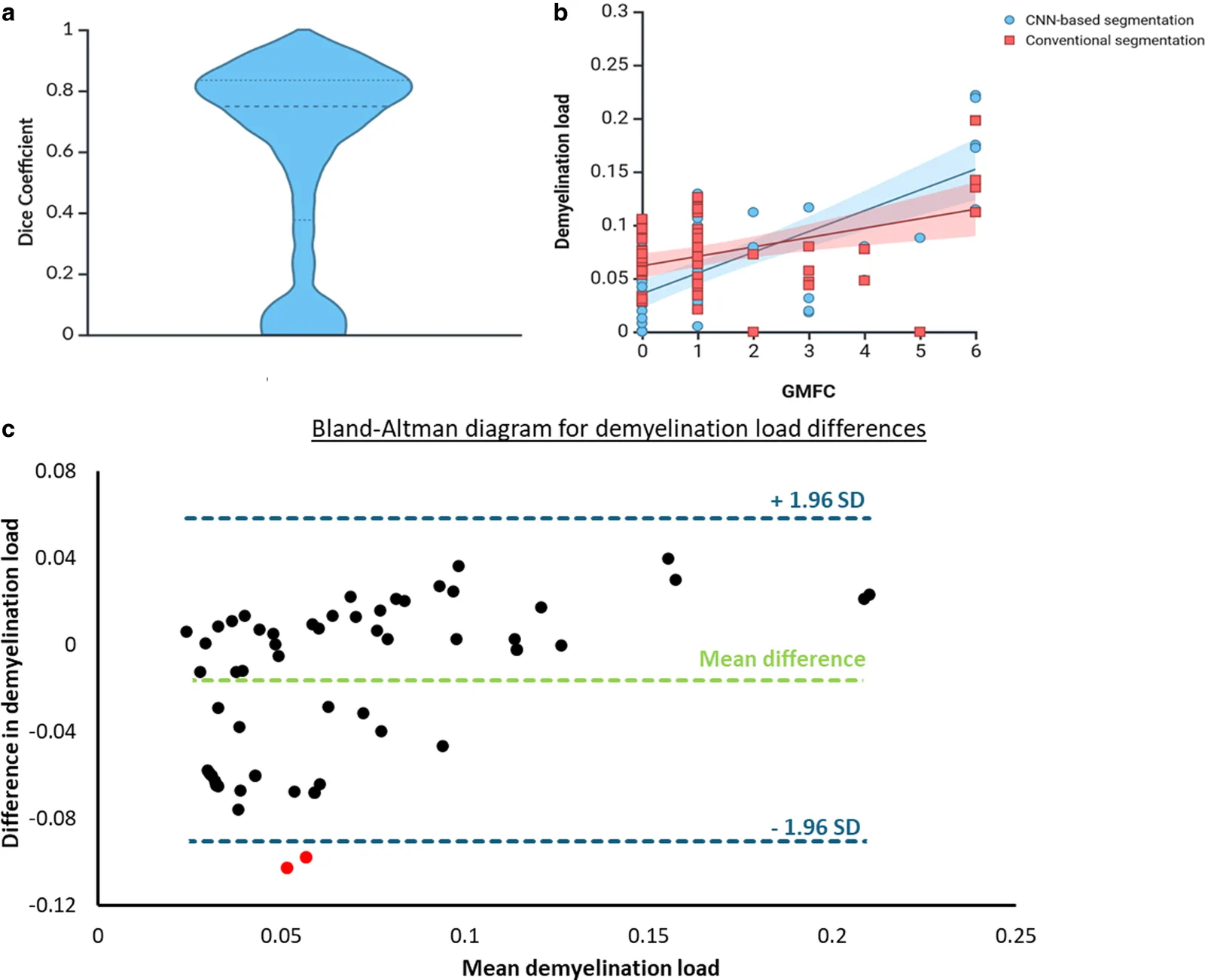
Performance of CNN vs. conventional segmentation on 2D T1-weighted images (**a**) Violin plot of Dice coefficients for CNN-based and conventional lesion segmentation. The width of the violin reflects the frequency of values; the dashed lines mark the 25th, 50th (median), and 75th percentiles. The lower quartile “tail” illustrates that, while most cases achieve high overlap (> 0.8), a subset shows markedly lower agreement. (**b**) Scatter plot of demyelination load versus GMFC-MLD score, comparing CNN-based (blue circles, blue regression line) and conventional (red squares, red regression line) segmentations. The CNN method shows slightly higher correlation with increasing disability levels. (**c**) Bland-Altman analysis showed high agreement with 2 datapoints falling out of the limits of agreement (3.6%). The CNN-based segmentation selected slightly less voxels as lesion compared to the conventional approach (median—0.0065)

Despite not being trained on 2D T1-weighted sequences, the CNN model performed in at least comparable accordance to the conventional segmentation. As expected the overall performance of both tested segmentation methods was less heterogeneous in the 2D T1-group than in the 3D T1-group. The mean dice coefficient was lower with 0.75 and standard deviation was higher with 0.32 (compared to 0.82 ± 0.2 in 3D T1). The 2D T1-group thus differed significantly from the 3D T1-group (*p* < 0.05). The violin plot in Fig. 6a demonstrates a vast majority of well according datasets, yet compared to Fig. 2a more data points fall in the lower DC range. Comparable to the results from 3D T1, no systematic over- or underestimation of lesion volume by the CNN-based model was observed in the Bland-Altman-Diagram (Fig. 6c). Regarding the Dice Coefficient, most outliers corresponded to scans with small volumes of T2-hyperintense white matter. With respect to clinical severity, the semiquantitative MLD MRI severity score showed a strong correlation with GMFC (r_S_ = 0.65, *p* < 0.001). CNN-derived demyelination load demonstrated a slightly lower but still robust association with GMFC (r_S_ = 0.56, *p* < 0.001), whereas the conventional volumetric measure showed only a weak correlation (r_S_ = 0.28, *p* = 0.036). A similar pattern was observed for radiological severity. CNN-derived demyelination load showed a stronger correlation with the MLD MRI severity score (r_S_ = 0.83, *p* < 0.001) compared with the conventional approach (r_S_ = 0.29, *p* = 0.03). Visual inspections supported the statistical finding that, in the case of 2D T1-weighted data, the conventional approach had incorrectly classified excessive white matter as demyelination load—maybe due to partial volume effects. Notably, small clusters located outside the typically confluent regions of demyelination were more frequently retained despite the application of the 200-voxel filter, which may not be sufficient for this modality.

## Discussion

This study demonstrates that a convolutional neural network (CNN)-based approach can reliably segment demyelinated white matter in MLD patients, showing substantial agreement with an established semi-automated reference method. The model performed robustly across heterogeneous MRI acquisition settings, including both 2D and 3D imaging protocols, and patient characteristics, with high spatial overlap (median Dice coefficient for 3D T1-images = 0.82, 0.75 for 2D T1-images), low segmentation bias, good correlation with clinical severity and pathophysiological plausible, steadily increasing lesion loads in longitudinal follow-up.

### Segmentation Accuracy and Influencing Factors

The overall segmentation agreement was good, with higher accuracy in 3D T1-weighted sequences compared to 2D T1 images. This discrepancy is expected, as 3D scans offer higher spatial resolution and contrast uniformity. The dice coefficient was most affected by low lesion burden, young age, and low GMFC scores—likely due to developmental factors such as incomplete myelination or greater anatomical variability, which challenge both segmentation methods. Importantly, in these cases the CNN-based approach more closely matched the visual impression than the conventional method, especially in the presence of low contrast or structural abnormalities.

Compared with recent deep-learning pipelines for multiple sclerosis lesion segmentation—which typically report Dice scores in the 0.75‑0.85 range and 5–10% outliers [16–18]—our CNN achieves comparable or even improved overlap on T1/T2 data. Unlike most MS models trained exclusively on adult FLAIR scans, it generalizes robustly across varied protocols and a markedly younger cohort, demonstrating that modern AI frameworks can be successfully adapted to challenging, heterogeneous pediatric leukodystrophy imaging.

### Outliers and Qualitative Findings

Visual inspection of segmentation outliers confirmed that most discrepancies stemmed from known pitfalls of conventional segmentation in neuroimaging, even more in a pediatric population—such as partial volume effects near the ventricles/CSF or misclassification of unmyelinated white matter. In contrast, the CNN model was less prone to oversegmentation in such areas across heterogeneous acquisition settings, including different field strengths, and showed greater spatial specificity. Importantly, no scan was excluded due to CNN-specific quality control failure that had not already failed quality control in the conventional pipeline. Moreover, low Dice scores were not randomly distributed but clustered in a small number of patients and remained relatively stable across their longitudinal scans, even when acquired on different MRI scanners. This suggests that certain anatomical or developmental conditions—such as early age, delayed or incomplete myelination, or ventricular deformation—create consistent challenges for conventional segmentation pipelines. These findings highlight the potential of deep learning approaches to better account for structural and maturational variability in pediatric MLD.

### Cross Modality Generalizability

Despite being trained exclusively on 3D data, the CNN model generalized well to 2D T1-weighted scans, albeit with moderately reduced Dice coefficients. This demonstrates its flexibility and potential applicability in real-world clinical settings where 2D acquisitions may still be common. Notably, small, isolated clusters outside typical lesion regions were more frequently retained in the conventional segmentation of low-resolution 2D scans. These likely reflect limitations of voxel-based filtering when applied to noisier or lower-contrast data. While increasing the filter threshold (e.g., above 200 voxels) might have reduced such spurious detections, predefined thresholds were deliberately kept constant across all analyses to preserve comparability between methods. This highlights the need for modality-specific adaptation in conventional pipelines, while the CNN model demonstrated better intrinsic robustness to such variations.

### Correlation with Clinical and Radiological Severity

Lesion volumes derived from both the CNN-based and the conventional segmentation methods showed significant correlations with motor impairment as assessed by the GMFC score. While the CNN-based model consistently yielded slightly stronger correlations in both cohorts, the differences were modest and did not indicate a substantial performance gap. Interestingly, the correlation was markedly stronger in the 2D T1-weighted dataset (r_S_ = 0.56) compared to the 3D T1-weighted dataset (r_S_ = 0.38), even though the CNN model was trained exclusively on 3D data. The reason for this discrepancy remains unclear. One possible explanation lies in the distribution of clinical severity within each group: in the 3D cohort, only a few patients had a GMFC score of 6, potentially limiting the range of observable clinical impairment and thus weakening the strength of the association. In contrast, the 2D group included a broader spectrum of disease severity, allowing for a clearer trend. Importantly, the correlation coefficients observed for the 2D data align well with previous studies on demyelination load and clinical function [7], suggesting that the CNN-based segmentation captures disease burden in a clinically meaningful way even in lower-resolution imaging. Notably, the semiquantitative MLD MRI severity score still demonstrated the strongest association with clinical severity in both 3D (r_S_ = 0.42) and 2D (r_S_ = 0.65) datasets.

In addition to clinical validation, CNN-derived demyelination load showed a stronger association with the established semiquantitative MLD MRI severity score than the conventional volumetric approach. For 3D T1-weighted data, the correlation with MLD MRI severity score was moderate but clearly higher for the CNN (r = 0.48) compared with conventional segmentation (r = 0.28). This difference was even more pronounced in the 2D cohort (CNN: r = 0.83; conventional: r = 0.29).

### Longitudinal Performance

In longitudinal datasets, both segmentation methods yielded plausible progression patterns, with volumetric changes consistent with natural disease evolution. The CNN approach showed no implausible fluctuations or outliers, supporting its suitability for longitudinal tracking. This aspect is crucial for applications in therapeutic monitoring, particularly in the context of emerging treatments like gene therapy.

### Clinical Implications and Future Applications

The CNN-based segmentation approach offers a promising alternative to conventional methods for quantifying demyelination in MLD. It demonstrated substantially reduced processing time and operator dependency, while maintaining strong concordance with semi-automated pipelines, even when evaluated across heterogeneous MRI sequence types and scanner acquisition parameters. Particularly in cases with subtle or ambiguous findings—such as early-stage disease or atypical patterns—the CNN model may provide more consistent results, helping to guide clinical interpretation. Furthermore, its applicability to both 3D and 2D T1-weighted scans makes it compatible with a broader range of clinical imaging protocols, including those used in resource-limited settings.

Importantly, the observed correlation between demyelination load and motor impairment supports its use as a potential imaging biomarker in future clinical trials or therapeutic monitoring. At the same time, other imaging tools like the visual semiquantitative MLD MRI severity score [2], DTI [19–21] and MR Spectroscopy [22, 23] showed good association with clinical status and therefore remain highly valuable and clinically meaningful parameters. In this context, CNN-derived demyelination load should be viewed not as a replacement, but as a complementary quantitative biomarker that adds continuous, observer-independent volumetric information to existing MRI-based outcome measures. It may meaningfully enrich multimodal imaging assessment alongside established biomarkers such as MR spectroscopy metrics, diffusion imaging parameters, and other microstructural imaging readouts [24], as well as visual rating scales. Future studies should systematically evaluate which combination of imaging parameters—and which specific quantitative metric—provides the greatest clinical relevance and sensitivity to change in different disease stages and therapeutic contexts. Given the increasing use of gene therapy in MLD (e.g., arsa-cel), such automated and scalable methods could assist in standardized evaluation of treatment response across centers. Nevertheless, human oversight remains imperative to ensure plausibility of automated results.

While the current model was trained on MLD data, the underlying architecture and principles are likely transferable to other leukodystrophies characterized by confluent white matter changes, such as X‑linked adrenoleukodystrophy (X-ALD). However, disease-specific adaptations and validations would be required before broader application, as lesion distribution, imaging characteristics, and age-dependent anatomical variability may differ.

### Limitations

This study has limitations. First, the ground truth was based on a conventional pipeline that itself is not error-free. Thus, segmentation discrepancies should not be interpreted as incorrect predictions per se—even though visual inspection in the very most cases suggested that the CNN model more accurately reflected the true extent of demyelination. Second, although the model performed well across 2D and 3D data, it was not explicitly trained on mixed-modal datasets. Future training using diverse input formats and scanner types may further improve robustness. Third, FLAIR imaging was not included in this study. While FLAIR may improve lesion conspicuity beyond early childhood—when myelination is more advanced—its diagnostic utility in younger individuals is limited by age-dependent myelination effects. In incompletely myelinated brains, the contrast between normal and demyelinated white matter is often reduced on FLAIR, making T2-weighted imaging more reliable for lesion detection. Moreover, FLAIR sequences were not consistently available across all sites and showed greater heterogeneity in acquisition parameters. Our approach therefore focused on routinely acquired T1- and T2-weighted sequences, which are available for practically all patients and form the basis of established semi-quantitative and volumetric scoring methods. Future studies incorporating standardized FLAIR or multi-contrast protocols may further enhance lesion characterization and model generalizability.

Fourth, the tissue probability maps used for conventional segmentation were derived from standard adult templates provided by CAT12/SPM12. While these offer robust segmentation in healthy adult populations, they are not specifically tailored to pediatric brains. As a result, anatomical deviations due to age-related differences or disease-induced brain changes in MLD may lead to systematic misclassification of tissue types, which in turn could bias the ground truth.

Finally, the cohort size—though substantial for a rare disease—remains limited. Larger, multicenter datasets are needed to validate generalizability and to train next-generation models with improved adaptability across ages and imaging protocols.

## Conclusion

In conclusion, this CNN-based segmentation approach provides a fast, scalable, and clinically relevant method for quantifying demyelination in MLD. It aligns closely with conventional methods, correlates with clinical severity, and is robust across modalities and time points. As precision imaging becomes integral to treatment monitoring in leukodystrophies, such tools will be instrumental in translating advanced MRI into routine clinical care.

## Data Availability

The datasets generated and/or analyzed during the current study are not publicly available due to patient privacy and institutional restrictions.
